# Experimental and Numerical Investigation of Dynamic Damage and Load Transfer of PBX Substitute Material under Low Velocity Impact

**DOI:** 10.3390/polym16091235

**Published:** 2024-04-28

**Authors:** Youcai Xiao, Qin Fu, Wanqian Yu, Chenyang Fan, Yu Zou, Yi Sun

**Affiliations:** 1 College of Mechatronic Engineering, North University of China, Taiyuan 030051, China; fuqing_nuc@163.com; 2 National Key Laboratory of Land & Air Based Information Perception and Control, Xi’an Modern Control Technology Research Institute, Xi’an 710065, China; hsywq812907@163.com; 3 Science and Technology on Electromechanical Dynamic Control Laboratory, Xi’an 710000, China; xyc374527@163.com; 4 No. 601 Institute of the Sixth Academy of CASIC, Hohhot 010076, China; 17835176096@139.com; 5 Departments of Astronautic Science and Mechanics, Harbin Institute of Technology, Harbin 150001, China; sunyi_hitgroup@163.com

**Keywords:** polymer-bonded sugar, shelled charge, Visco-SCRAM model, crack, VUMAT

## Abstract

The accidental initiation of explosives under mechanical loads has caused numerous catastrophic events. Therefore, the dynamic damage behavior of confined polymer-bonded explosives (PBXs) must be assessed to improve their practical applicability. In this study, polymer-bonded sugar (PBS) materials were prepared using a novel agglomerate to develop a PBX substitute material with enhanced experimental safety. The mechanical properties of the PBS shell were evaluated using a dynamic compression test, which revealed that the compression response of the shell was affected by the strain rate. A low-velocity impact experiment was performed to investigate the dynamic damage and load transfer characteristics of the PBX substitute. A constitutive model was developed to characterize the mechanical response of PBS subjected to high strain rates, and implementing this model in ABAQUS ensured successful prediction of the damage evolution process associated with PBS. Simulation results indicated that the PBS specimen was primarily damaged around its center while sliding friction was dominant near the center during pressure application. Notably, different stress states result in distinct crack growth velocity histories along the axial direction, with the damage ratio progressively decreasing toward regions closer to the impact surface.

## 1. Introduction

Polymer-bonded explosives (PBXs) are particulate composites containing energetic materials (such as HMX and RDX) embedded in a polymer binder, which have been widely used in the modern military, aerospace industry, and other fields [1,2]. During production, storage, transportation, etc., minor impacts (dropping, collision, friction) on PBX can cause microcracks, micro-holes, and additional damage [3,4], which can lead to accidental ignition and result in economic losses [5,6]. Therefore, understanding the damage behavior of PBX under mild-impact loading is crucial for ensuring its safe utilization.

Numerous attempts have been undertaken to investigate the mechanical behavior and failure mechanisms of energetic substances through experimental approaches [7,8,9,10]. Rae et al. [11,12] investigated the deformation and failure behaviors of PBX9501 through the Brazilian test and optical microscopy. Their findings revealed that failure paths tend to circumvent regions with fine fillers and binders, thereby preferentially propagating along the elongated straight edges of the explosive filler. The microstructural damage progression involves detachment of large particles from the matrix, splitting of grains, cracking in binders, and initiation and growth of voids. Chen et al. [13] investigated the damage behavior of sectional PBX charges during penetration; their findings revealed that the most pronounced microcrack damage occurred at the head and tail of the projectile. Additionally, the charge’s impact sensitivity substantially increased. Gao et al. [14] tested the penetration strength of the ground-drilling projectile for a target to investigate the impact of the charge structure and buffer material on the charge stability. Subsequent examination of the retrieved charge structure revealed the presence of cracks within the charge, and a detached ring was observed in the contact region between the charge and shell casing at the rear end. Chen et al. [15] developed a substitute PBX material by combining sugar and a novel agglomerant to produce polymer-bonded sugar (PBS) materials. The dynamic test was conducted using the split Hopkinson pressure bar (SHPB) technique at varying impact velocities, while micromechanics was used to analyze the nucleation of cracks in PBS. Results suggest that the microstructure of this material can be altered by inducing microcracks via dynamic tensile stress on surface defects. Li et al. [16] investigated the dynamic damage mechanisms and non-shock initiation of PBX1314 during penetration through a combination of experiments and simulations. The experimental approach involved launching steel projectiles filled with PBX1314 to penetrate concrete targets. The obtained results revealed occurrences of non-shock initiations at the tail surface of PBX1314, which was accompanied by mechanical damage observed in both the tail and middle sections of the material. At present, the mechanical damage characteristics of charges are primarily investigated through Hopkinson and penetration experiments. The macroscopic fragmentation of shells observed in penetration experiments reveals vulnerable areas of ammunition during penetration; however, it cannot be used to accurately depict the actual stress state of the charge.

In addition to conducting experimental research, numerous theoretical studies have elucidated the mechanical behavior and damage mechanism of PBXs [17,18,19,20,21]. Dienes [22,23] initially formulated the statistical crack mechanical model (SCRAM) to account for various characteristics (such as crack opening and shearing) in the growth and coalescence of multiple penny-shaped cracks; the primary objective was to explore the impact sensitivity in PBX composites. Based on SCRAM, Bennett et al. [24,25] used statistical crack mechanics to develop a general Maxwell element by taking into account five Maxwell elements and a constant spring; this model has been widely used to simulate the dynamic damage behavior of different types of PBXs [26,27,28]. The predictive capabilities of the model were demonstrated by using it to forecast the occurrence of hot spots in PBX9501 during dynamic impacts. Remarkably, the simulated outcomes align well with experimental findings on non-shock ignition conducted by Asay et al. [29,30]. Recently, crack damage has been modeled using the dominant crack algorithm (DCA), which effectively captures both open and closed cracks. Open cracks can either occur via mode-I opening or through a combination of shear and opening modes. Meanwhile, closed cracks include friction-locked cracks and pure shear cracks. Yang et al. [31,32] examined the formation of hotspots in PBX under various impact conditions by integrating DCA, viscoelasticity, and plastic elements, and the damage distribution of PBX9501 in the experiment was accurately predicted. Labarbera et al. [33] studied crack nucleation, propagation, and hot spot formation of RDX/Estane energy aggregates under dynamic loads. Dai et al. [34] elucidated the damage mechanism observed in PBX substitute materials when subjected to compressive loads, and the primary cause of damage was attributed to the initial formation of pores and cracks resulting from granule fragmentation. Under strain, both granule size and porosity decrease while damage levels increase. Liu et al. [35] investigated the development of microcracks under tension and compression and elucidated the formation mechanism of friction-based hotspots by using a dynamic damage viscoelastic model. The study successfully captured the occurrence of cracking in both tension and compression scenarios while accurately simulating the damage morphology. However, the accurate prediction of the failure of PBXs under complex mechanical stress necessitates a constitutive model accounting for the dominant damage.

Theoretical models provide valuable insights into the damage mechanisms of PBX under impact loadings. The corresponding calculations accurately predict the mechanical characteristics of PBXs in experimental scenarios. However, only simple impact loads, such as uniaxial compression at constant strain rates, have been considered in the aforementioned studies. In real-world engineering scenarios, PBX structures are subjected to complex loads. Moreover, the aforementioned studies have focused on the pressed energetic material PBX9501. Furthermore, few studies have reported on dynamic damage and load transfer in the context of PBXs, which are extensively used in military weapons.

In this study, a PBS shell was employed as a PBX substitute material to investigate its mechanical response and damage evolution mechanisms under impact loading conditions. The load transfer experiment was designed by utilizing a first-stage light gas gun to explore the dynamic damage propagation and axial load transfer of PBS. We investigated the propagation behavior of the PBS shell charge under impact loading conditions and analyzed the experimental findings. The damage constitutive relationship of PBS was studied, and the numerical simulations supported the applicability of the established constitutive model. Based on the outcomes of the numerical simulation, the evolution of PBS charge damage under low-velocity impact was examined.

## 2. Experiment

### 2.1. Materials and Specimen

**Figure 1:**
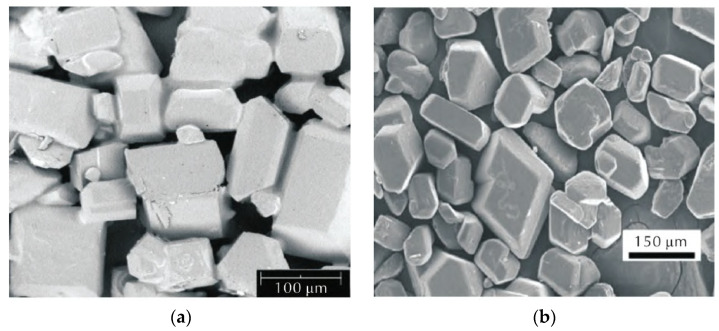
SEM micrographs of (**a**) sugar granule and (**b**) HMX granule.

PBS comprised sugar granules and a polymer binder, with a weight ratio of 90 parts sugar to 10 parts binder [27]. Additionally, it exhibited a density of 1.49 g/cm^3^. The PBS composition is shown in Table 1. The dual-carbon white sugar particles were used as a substitute for the energetic HMX particles in PBX. Figure 1 illustrates the microstructure of both the white sugar particles and HMX particles, which are predominantly polyhedral in shape, with well-defined edges and corners closely resembling those of HMX particles.

**Figure 2:**
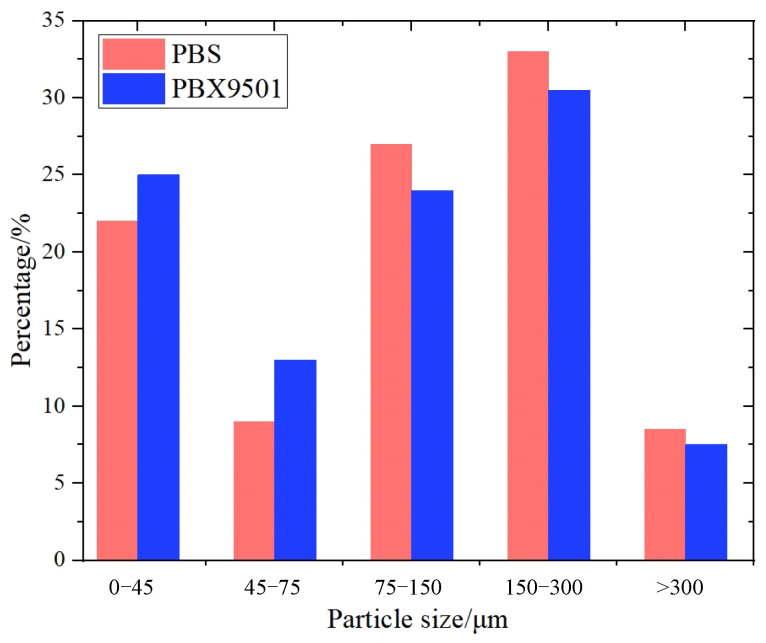
Histogram depicting the distribution of particle diameters.

The mechanical properties and damage mode of PBXs are notably influenced by the size distribution of energetic particles, thereby rendering the particle size distribution of substitute materials for PBXs crucial. The mechanical properties and damage mode of PBXs are considerably affected by the energetic particle size distribution, making the particle size distribution of PBX substitute materials another critical factor. However, the particle size distribution of white sugar is broad and spans from a few micrometers to several hundred micrometers. Processed sugar particles were sequentially sieved through screens with mesh sizes of 60, 100, 200, and 325. The selected particles were weighed using an electronic scale, and their mass ratio was determined and compared with the PBX component ratio depicted in Figure 2. As indicated by the results, the particle size distribution of the substitute material exhibited a high degree of consistency with that of PBX.

**Figure 3:**
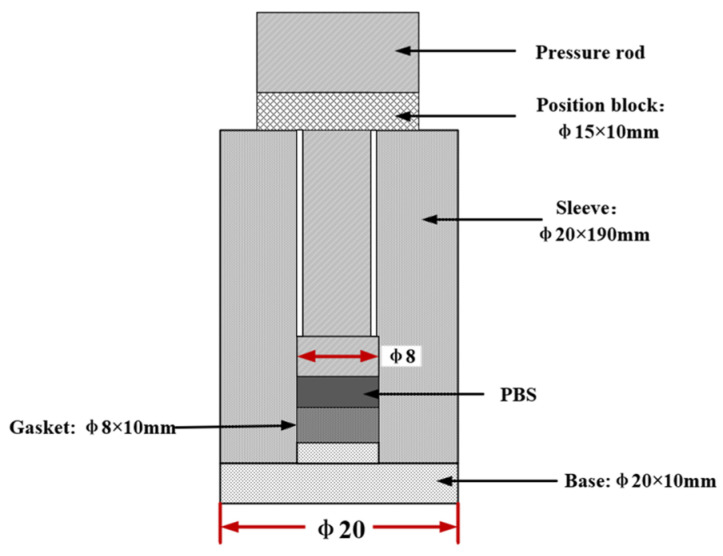
Schematic of the mold. The symbol Φ represents the diameter of the apparatus.

**Figure 4:**
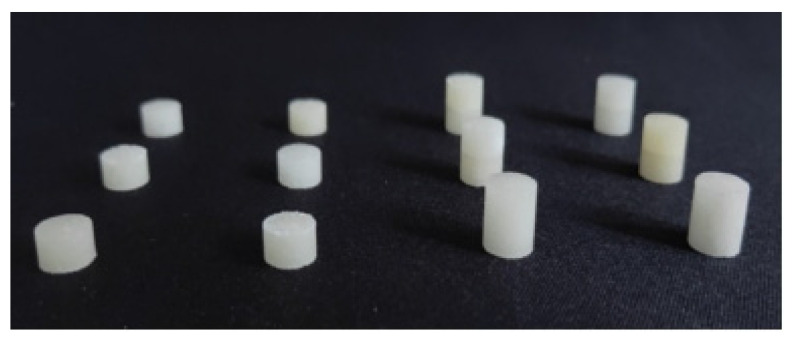
Samples after demolding.

To ensure that the PBS sample was prepared properly, the following steps were taken. Firstly, granulated sugar was subjected to ultrasonic treatment in ethanol without water. Subsequently, processed sugar was filtered using a suction filtration apparatus and then placed in a vacuum freeze dryer at −10 °C. This procedure was performed to improve the flowability of the sugar, thus better simulating energetic particles. Figure 3 shows the shape and size of the mold. Secondly, hydroxyl-terminated polybutadiene (HTPB) and a plasticizer (dioctyl sebacate, DOS) were mixed in a stirrer and stirred for 20 min. After adding the curing agent (tolylene-2,4-diisocyanate, TDI), stirring was performed for 10 min more. Upon completion of the stirring process, the mixture was cured for 24 h at 70 °C. Finally, a portion of the cured mixture was cold pressed at 340 MPa in a pre-designed mold to fabricate the required experimental specimens. The resulting samples are shown in Figure 4.

**Figure 5:**
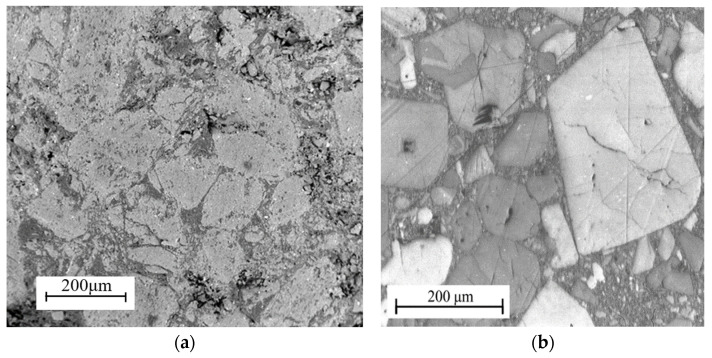
SEM micrographs of (**a**) PBS and (**b**) PBX.

The morphology of PBS and PBX, as well as the microstructure of sugar and HMX particles, is depicted in Figure 5. Notably, a majority of the sugar and HMX particles exhibit polyhedral shapes with distinct edges and corners. The particle content is relatively high, while the initial non-uniform microcracks and micro-pores are distributed throughout both the particles and binder.

### 2.2. Dynamic Test of PBS Samples

**Figure 6:**
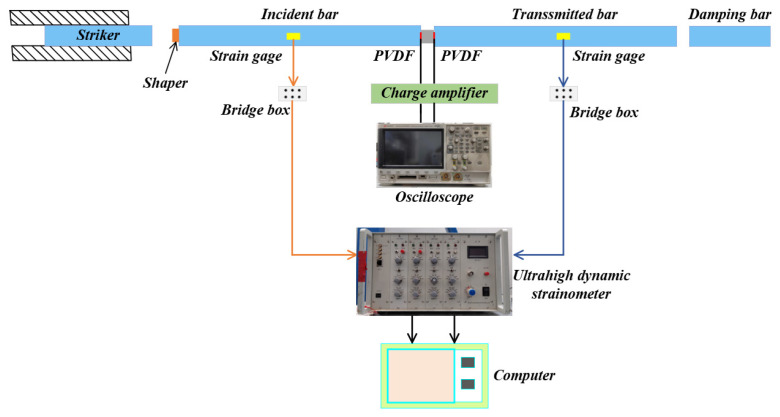
Dynamic compression device based on SHPB.

Compression experiments were conducted on the PBS specimen using a modified SHPB setup (Figure 6). The SHPB system comprises a gas gun that propels the striker bar toward the incident bar, which generates an elastic compression wave that travels through the incident bar and reaches the specimen. In cases where the wave impedance of the specimen is lower than that of the bars, an elastic tensile wave reflects back into the incident bar and an elastic compression wave passes through to the transmission bar. The diameter and length of both the striker and transmission bars are 12 mm and 1500 mm, respectively. Furthermore, aluminum bars with a density of 2700 kg/m^3^ and Young’s modulus of 73 GPa were used in this study. A layer of high-purity petroleum jelly was applied between the sample and rod surfaces to minimize frictional effects during testing. The PBS specimen has a diameter and thickness of 8 mm and 4 mm, respectively.

The duration of the loaded waveform was increased using a circular lead sheet to ensure that multiple internal reflections occurred within the specimen to achieve stress equilibrium. The bars were supported by well-lubricated tripods, which allowed free movement with negligible friction effects. Both the incident and transmitted bars were equipped with a pair of strain gauges that were mounted in pairs at four specific locations to form a half-bridge circuit, thereby enabling the measurement of axial strain caused by the propagation of uniaxial elastic stress waves at each location. The incident wave ($\varepsilon_{i}$), reflected wave ($\varepsilon_{r}$), and transmitted wave ($\varepsilon_{t}$) measurements were obtained by utilizing resistance strain gauges that were symmetrically positioned on the cylindrical surfaces of both the incident and transmitted bars. The specimens’ stress (σ), strain (ε), and strain rate ($\dot{\varepsilon }$) are provided below [36,37]:

(1) $$\varepsilon =\frac{2C_{0}}{L_{s}}\int_{0}^{t}\varepsilon_{r}d\tau$$

(2) $$\dot{\varepsilon }=\frac{2C_{0}}{L_{s}}\varepsilon_{r}\left(t\right)$$

(3) $$\sigma =\frac{A_{0}}{A_{s}}E_{0}\varepsilon_{t}\left(t\right)$$

where *C*_0_ denotes the velocity of longitudinal waves in the bars, *L_s_* represents the initial length of specimens, *E*_0_ signifies Young’s modulus of the bars, and *A*_0_ and *A_S_* denote the cross-sectional areas of the bars and specimens, respectively.

### 2.3. Load Transfer Test

**Figure 7:**
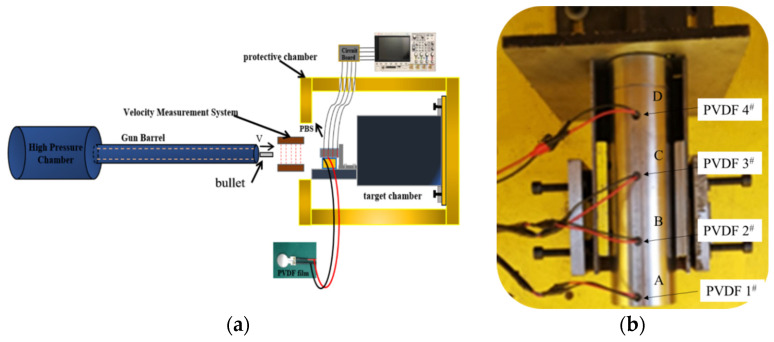
(**a**) Schematic of the experimental setup; (**b**) the test specimen and PVDF stress sensor. The dashed yellow line represents the inner diameter of the light gas gun, while the dashed red line denotes the laser velocity measurement light path. The # symbol indicates the number of PVDF sensors, and A, B, C, D represent their respective positions.

Figure 7a shows that the experimental setup primarily consists of a light gas gun, bullet, and velocity measurement chamber. The high-pressure nitrogen gas chamber within the light gas gun propels the bullet at high speeds through the gun barrel. The laser velocity measurement chamber quantifies the projectile’s velocity as it enters the target chamber and collides with the specimen, producing a stress wave that diminishes in the propagation direction. The polyvinylidene fluoride (PVDF) pressure sensor affixed to the specimen captures and records this pulse signal.

The test specimen and PVDF stress sensor are depicted in Figure 7b. The PBS sample had a diameter and length of 50 mm and 200 mm, respectively. Meanwhile, the casing (made of 4340 steel) was 60 mm in diameter and 210 mm in length. The projectile was a cylindrical piece made from the same material (4340 steel), with a diameter and length of 20 mm and 100 mm, respectively. Polyvinylidene fluoride (PVDF) sensors were used to measure the impact pressure on the samples. Four holes with a diameter of 7 mm were drilled on the outer side of the shell at intervals of 50 mm to extract wires from the PVDF pressure sensor. The PVDF pressure sensor and the charge amplification circuit were connected to the KEYSIGHT DSOX3012T series oscilloscope for real-time monitoring of internal pressure within the PBS specimen.

Under impact loads, the discharging capacity of the PVDF sensor was obtained by integrating the current flowing through the resistance *R* [38,39]:

(4) $$q(t)=\int_{0}^{t}\frac{U(\tau )}{R}d(\tau )$$

The relationship between stress pulses and discharge charge is as follows:

(5) $$\sigma (t)=\frac{q(t)}{S\times d_{33}}$$

where *d*_33_ is the piezoelectric constant and *S* is the effective area of the PVDF stress sensor. The relationship between stress and test voltage can be derived by combining Equations (4) and (5). The piezoelectric constant of the PVDF stress sensor is 43.94 pC/N.

## 3. Experimental Analysis and Discussion

### 3.1. Compressive Properties of PBS at High Strain Rate

**Figure 8:**
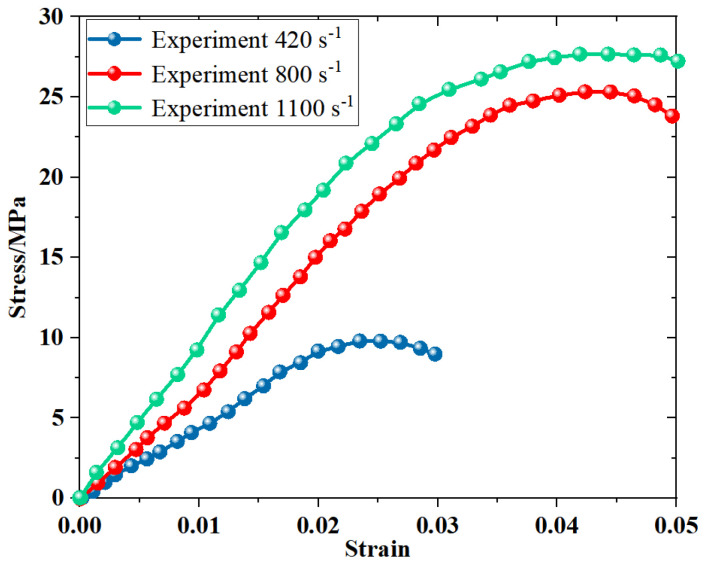
Stress−strain curves of the PBS under dynamic compressive loading.

The dynamic compression stress-strain curves of PBS (Figure 8) indicate a prominent strain rate effect, wherein the compressive strength and modulus of PBS increase substantially with increasing strain rates. These findings further corroborate that the properties of PBS closely resemble those of PBX under specific conditions.

### 3.2. Load Transfer Behavior of PBS Shell

**Figure 9:**
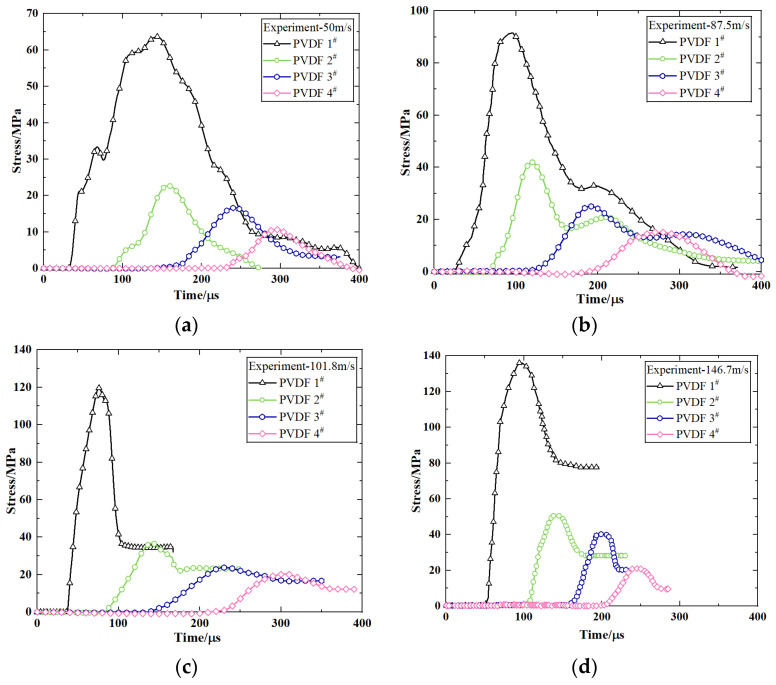
Time−pressure curves for various impact velocities: (**a**) 50 m/s, (**b**) 87.5 m/s, (**c**) 101.8 m/s, and (**d**) 146.7 m/s. The “#” symbol indicates the number of PVDF sensors.

The time-pressure signals measured using PVDF at impact velocities of 50 m/s, 87.5 m/s, 101.8 m/s, and 146.7 m/s are presented in Figure 9. The initial intervals of the pressure history curves remain relatively consistent across different positions (~60 µs), thereby indicating a consistent propagation of stress waves within the medium and a stable wave velocity. The ascending segment of individual curves exhibits minor amplitude oscillations, which may be attributed to the wave impedance mismatch between the PVDF pressure sensor and PBS material employed for measurement purposes. Despite the occurrence of reflections at interfaces, these phenomena exert negligible influence on peak pressures.

**Figure 10:**
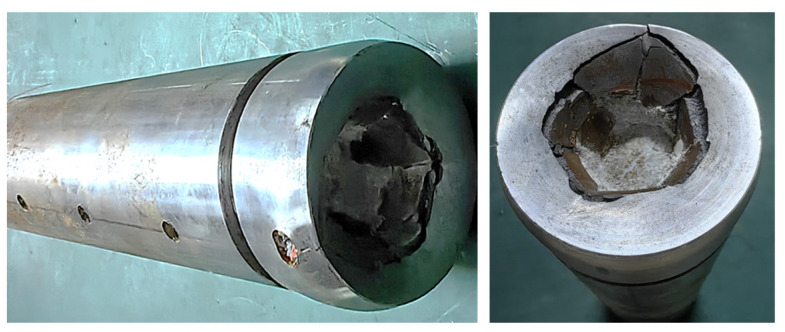
PBS projectile sample after being impacted at a strike velocity of 146.7 m/s.

The axial propagation of stress wave is illustrated in Figure 9 and demonstrates a distinct characteristic of exponential decay. The stress wave is initiated at the leading edge of the projectile and undergoes significant attenuation within the first 50 mm, which is followed by gradual stabilization beyond 150 mm. Although an increase in impact velocity results in a higher pressure on the projectile head, the peak value at 150 mm diminishes substantially with reference to its initial magnitude. Moreover, the peak plateau becomes more pronounced with increasing propagation distance. Figure 10 illustrates the PBS projectile sample after it impacted at a velocity of 146.7 m/s.

**Figure 11:**
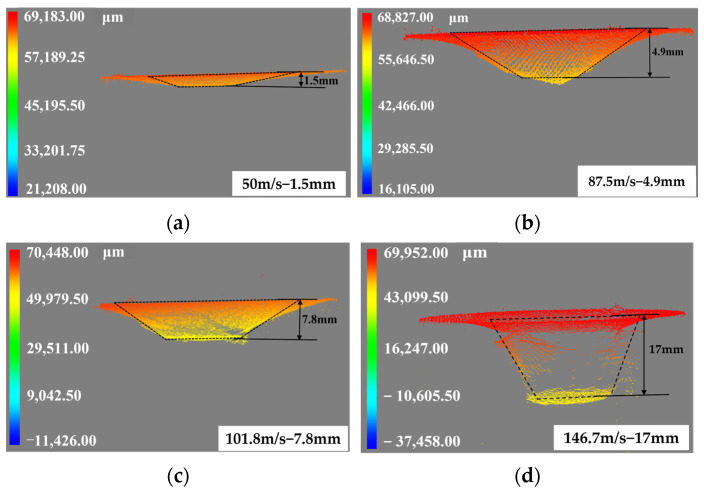
Scans of penetration pits under different velocities: (**a**) 50 m/s, (**b**) 87.5 m/s, (**c**) 101.8 m/s, and (**d**) 146.7 m/s.

The penetration depths at impact velocities of 50 m/s, 87.5 m/s, 101.8 m/s, and 146.7 m/s are illustrated in Figure 11., with the penetration depth increasing in proportion to the impact velocity.

## 4. Theoretical Analysis

### 4.1. Constitutive Model Formulations

The model formulation presented in this study is based on the research conducted by Addessio and Johnson [40], which incorporates a generalized Maxwell model (GMM). In this study, we utilized an isotropic constitutive relationship to describe the damage response of PBS. Throughout the deformation process, it is assumed that the distribution of cracks remains stochastic and follows an exponential size distribution.

The rate of deviatoric stress in the Visco-SCRAM model is calculated as follows:

(6) $${\dot{S}}_{ij}=\frac{2G_{total}{\dot{e}}_{ij}-\sum_{n=1}^{N}\frac{S_{ij}^{\left(n\right)}}{\tau^{\left(n\right)}}-3\frac{1}{a}{\left(\frac{c}{a}\right)}^{3}\dot{c}S_{ij}}{1+{\left(\frac{c}{a}\right)}^{3}}$$

where $G_{total}=\sum_{n=1}^{N}G^{\left(n\right)}$, *c* is the average crack radius, *a* is the initial flaw size, ${\dot{e}}_{ij}$ is the deviatoric strain rate, and $S_{ij}^{\left(n\right)}$ is the deviatoric stress of the nth Maxwell component.

The deviatoric stress rate in each Maxwell element is determined as follows:

(7) $${\dot{S}}_{ij}^{\left(n\right)}=2G^{\left(n\right)}{\dot{e}}_{ij}-\frac{S_{ij}^{\left(n\right)}}{\tau^{\left(n\right)}}-\frac{G^{\left(n\right)}}{G_{total}}\left[{\left(\frac{c}{a}\right)}^{3}{\dot{S}}_{ij}+3\frac{1}{a}{\left(\frac{c}{a}\right)}^{3}\dot{c}S_{ij}\right]$$

Typically, Equation (7) is written as follows:

(8) $${\dot{S}}_{ij}^{\left(n\right)}=2G^{\left(n\right)}{\dot{e}}_{ij}\left(1-\xi \right)-\frac{S_{ij}^{\left(n\right)}}{\tau^{\left(n\right)}}+\frac{G^{\left(n\right)}\xi }{G_{total}}\sum_{n=1}^{N}\frac{S_{ij}^{\left(n\right)}}{\tau^{\left(n\right)}}-3\frac{G^{\left(n\right)}\xi }{G_{total}}\frac{\dot{c}}{c}\sum_{n=1}^{N}S_{ij}^{\left(n\right)}$$

where $\xi ={\left(\frac{c}{a}\right)}^{3}/\left[1+{\left(\frac{c}{a}\right)}^{3}\right]$.

Dienes [22] reported that the propagation rate of cracks in the Visco-SCRAM model was influenced by stress intensity. However, stress intensity alone does not adequately represent actual microcrack growth characteristics. To address this limitation, Zuo et al. [23] and Dienes et al. [41] developed a DCA that accounts for the critical energy-release rate as a driving force for microcrack growth.

When the stress intensity is below a critical value, the crack velocity $\dot{c}$ can be determined as follows:

(9) $$\dot{c}=v_{R}{\left(\frac{g\left(\sigma ,n,c\right)}{g_{1}}\right)}^{m}\begin{array}{cc} & g\left(\sigma ,n,c\right)<g_{0} \end{array}$$

where $g\left(\sigma ,n,c\right)$ denotes the rate at which energy is released, *c* signifies the dimensions of the crack, *n* indicates the orientation of the microcrack surface, *V_R_* represents the highest velocity attained by the crack, and *m* corresponds to a model parameter typically ranging from 5 to 10.

When the stress intensity is high, the crack velocity $\dot{c}$ is determined as follows:

(10) $$\dot{c}=v_{R}\left[1-\frac{g_{c}}{g\left(\sigma ,n,c\right)}\right]\begin{array}{cc} & g\left(\sigma ,n,c\right)\geq g_{0} \end{array}$$

where $g_{c}=2\gamma$ represents the critical energy-release rate. The constant parameters $g_{0}$ and $g_{1}$ can be written as follows:

(11) $$g_{0}=\left(1+\frac{1}{m}\right)g_{c}$$

(12) $$g_{1}={\left(1+m\right)}^{\frac{1}{m}}\left(1+\frac{1}{m}\right)g_{c}$$

Intrinsically, Equations (9) and (10) reflect a transition from slow to fast crack growth. The transition point is continuous:

(13) $$g\left(\sigma ,n,c\right)=\frac{4\left(1-v\right)}{\pi \left(2-v\right)}\frac{f\left(\sigma ,n\right)c}{G}$$

where *ν* represents Poisson’s ratio. The stress function $f\left(\sigma ,n\right)$ varies depending on the types of microcrack evolution. Five different cracks are observed in the $\sigma_{1}-\sigma_{3}$ plane: pure tension cracks $-\left(1-v\right)\leq r\leq 1$, mixed tensile and shear cracks $-1\leq r<-\left(1-v\right)$, pure shear cracks $-{\left(\sqrt{\mu_{s}^{2}+1}+\mu_{s}\right)}^{2}\leq r\leq -1$, cracks formed due to shear and friction ${\left(\sqrt{\mu_{s}^{2}+1}+\mu_{s}\right)}^{4}<r^{2}<+\infty$, and friction-locked cracks $1\leq r\leq {\left(\sqrt{\mu_{s}^{2}+1}+\mu_{s}\right)}^{2}$ (where $r=\sigma_{3}/\sigma_{1}$).

Hence, it is preferable to characterize the propagation of cracks using the energy-release rate $g\left(\sigma ,n,c\right)$, which allows for computational determination of crack growth velocities irrespective of whether the crack is open or closed.

The specimen undergoes deterioration due to the growth of microcracks. As the radius of these microcracks expands, the damage level increases. This damage can be evaluated on a scale from 0 to 1 using the following expression: $D=c^{3}/\left(a^{3}+c^{3}\right)$ [42].

### 4.2. Numerical Algorithm and Model Verification

The proposed model was implemented using the user material subroutine (VUMAT) within the commercially available software package ABAQUS 2016. The numerical algorithms employed for this purpose can be summarized as follows:

(I) The system of equations in Equation (8) consists of n differential equations, which were solved using the single-step fourth-order Runge-Kutta method. The algorithm is strain driven. The solutions of stress and internal variables are required based on a known state of stress ($S_{ij}^{old}$) and internal variables ($c^{old}$ and ${\dot{c}}^{old}$). Finally, the trial stress state ($S_{ij}^{new}$) can be calculated as follows: $S_{ij}^{new}=S_{ij}^{old}+\Delta S_{ij}$.

(II) Based on the stress functions $f\left(\sigma ,n\right)$ under various conditions, Equations (9) and (10) were employed to calculate the crack growth velocity under different stress conditions.

(II) The average crack radius c at time t+Δt was calculated by determining the stress $S_{ij}$ at time t By solving Equation (8), this algorithm enables the acquisition of stress components at time t+Δt.

**Figure 12:**
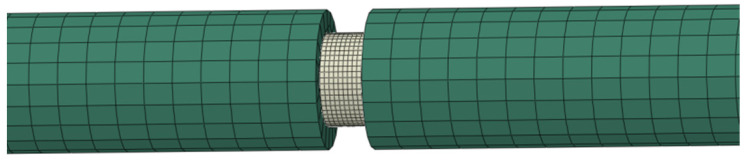
SHPB finite element model.

**Figure 13:**
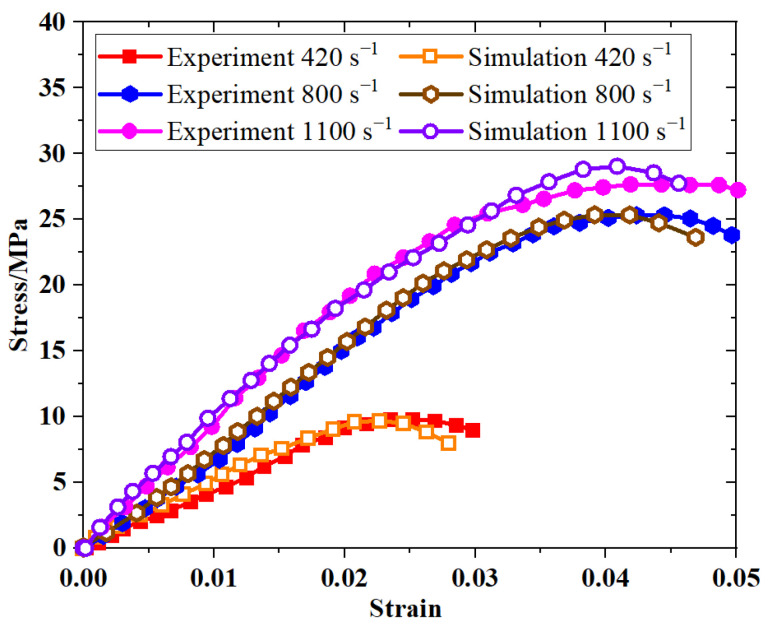
Simulated and experimentally obtained stress−strain curves of uniaxial dynamic compression in PBS.

Based on the experimental findings, ABAQUS software was employed to develop a finite element model (FEM) to validate both the damage constitutive model and numerical algorithm for PBS. Figure 12 depicts the FEM utilized during the dynamic compression test. All components were meshed using reduced integration 3D elements, specifically the C3D8R element from the ABAQUS element library. The grid convergence of the PBS specimens was evaluated using three different mesh sizes: 0.2, 0.5, and 1 mm. The peak stress obtained with the 0.5-mm mesh was only 1.7% higher than the peak value achieved with the 0.2-mm mesh, indicating that a mesh size of 0.5 mm is adequate for reliable predictions. Therefore, the PBS specimen was discretized with a mesh size of 0.5 mm, whereas the mesh size implemented for both the incident bar and transmission bar was 3 mm. The parameter values for PBS are presented in Table 2 and Table 3 [43]. Figure 13 illustrates the stress-strain curves obtained from both testing and simulation. The numerical results exhibit excellent agreement with the experimental findings, thereby confirming the validity of the constitutive model.

### 4.3. Analysis of Damage Behavior and Load Transfer of Shell PBS

#### 4.3.1. Load Transfer Simulation of Shell PBS

**Figure 14:**
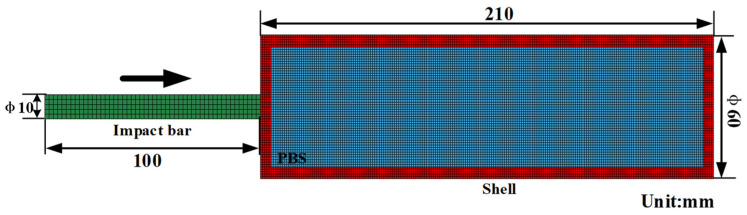
Finite element meshes. The symbol Φ represents the diameter of the apparatus, while the arrow indicates the direction of impact for the impact bar.

The proposed constitutive model was used to simulate the load transfer experiment under various velocities. The computational model schematic is illustrated in Figure 14. Specifically, the shell meshes are characterized by dimensions of 1 mm. For impact bar meshes, elements possess edges measuring 2 mm in both axial and radial directions. The PBS specimen was discretized with a mesh size of 1 mm. Table 4 provides the Johnson-Cook parameters for steel, with both the material model and its parameters being adopted from a previous study [43].

**Figure 15:**
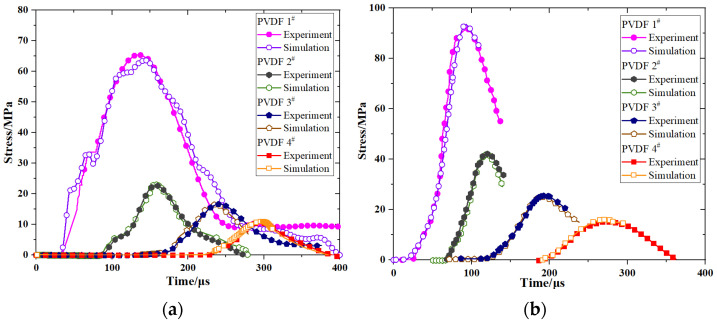

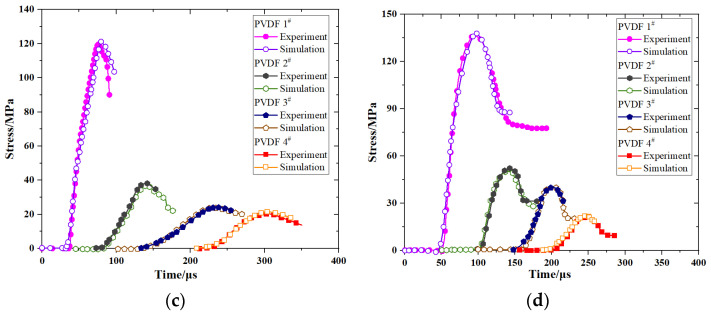
Comparison between experimental and simulated data at different velocities: (**a**) 50 m/s, (**b**) 87.5 m/s, (**c**) 101.8 m/s, and (**d**) 146.7 m/s. The symbol “#” represents the numerical value of PVDF.

**Figure 16:**
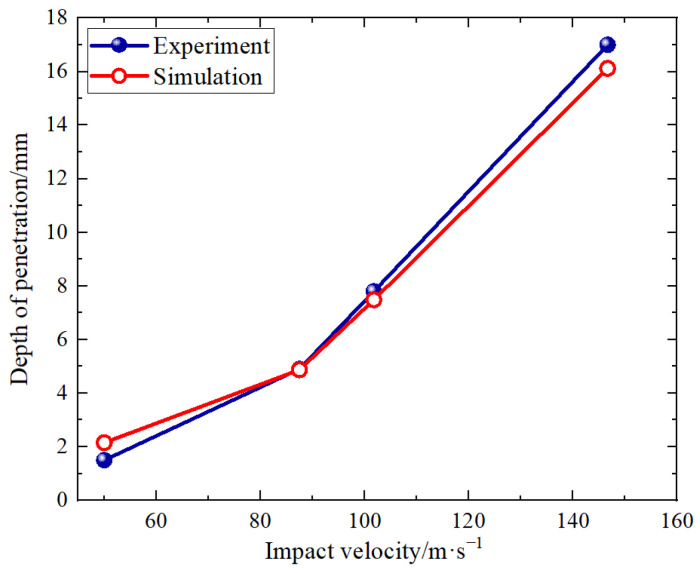
Comparison of simulated and experimental penetration depths.at different velocities.

The experimental and simulation results were compared by integrating the constitutive model into ABAQUS (Figure 15). The excellent agreement between the results validates the proposed constitutive model. Furthermore, Figure 16 illustrates the displacement curve at the midpoint of the impacted surface, which exhibits a close correspondence with the experimental penetration depth; this observation further confirms the accuracy of our constitutive model and subroutine.

#### 4.3.2. Pressure and von Mises Stress of PBS

**Figure 17:**
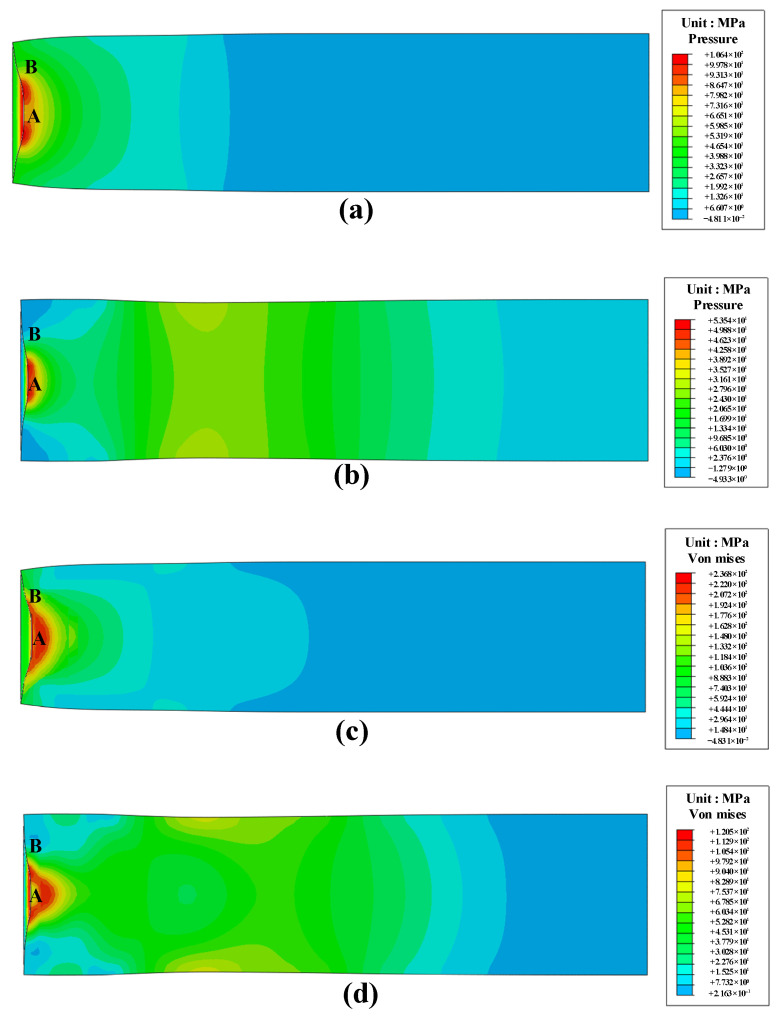
Pressure distribution of 87.5 m/s at (**a**) 70 µs, and (**b**) 140 µs; Von Mises stress distribution of 87.5 at m/s (**c**) 70 µs, and (**d**) 140 µs. The A and B denote the locations of compression within the specimen.

**Figure 18:**
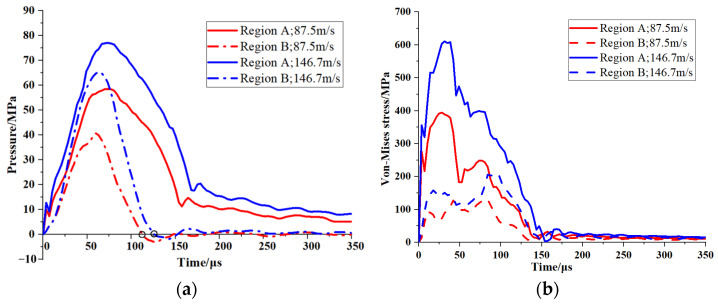
(**a**) Pressure and (**b**) von Mises stress profiles of regions A and B (Figure 15) at impact velocities of 87.5 m/s and 146.7 m/s.

Upon impact with the PBS target, the incident wave diverges and propagates through the PBS sample, resulting in a complex array of stress states. Regions A and B within the PBS were selected for the analysis (Figure 17) to fully capture the stress distribution. The contours of pressure and von Mises stress are shown in Figure 17 for *t* = 70 µs and 140 µs at an impact velocity of 87.5 m/s. The results depicted in Figure 17a,b demonstrate the propagation of a quasi-semicircular compressive wave through the sample after the initial impact, with the maximum pressure being 57 MPa at 70 µs. Subsequently, rarefaction waves emerge at the sample boundary and propagate into the compressive region, thus reducing the pressure amplitude that exhibits a maximum value of 25 MPa at 140 µs. The triangular-shaped zone beneath the rod exhibits high von Mises stress (~400 MPa) at 70 µs (Figure 17c,d). At 140 µs, this zone diminished and converged toward the axis line of the sample due to rarefaction effects. To further investigate wave propagation in the PBS sample, Figure 18a,b depict the pressure and von Mises stress histories at regions A and B, corresponding to impact velocities of 87.5 m/s and 146.7 m/s, respectively. The pressure curves depicted in Figure 18a demonstrate the emergence of a zone in regions A and B that experiences tension at 126 µs and 110 µs, respectively. This observation suggests the occurrence of rarefaction waves.

The results depicted in Figure 18a demonstrate that at an impact velocity of 87.5 m/s, the pressure ratio in regions A and B remains at approximately 1.35, indicating a greater influence of pressure in region A compared to region B. Moreover, Figure 18b illustrates that the von Mises stress ratio for the same region and velocity is ~2.5, thus suggesting a significant concentration of stress in region A. Interestingly, similar conclusions are achieved at a velocity of 146.7 m/s; that is, an even higher ratio of von Mises stresses is observed between the two regions. This discrepancy can be attributed to the significantly higher level of stress on the shell and its propagation speed compared to the PBS material, resulting in transverse rarefaction waves entering the PBS and causing increased internal stresses within it. This analysis reveals that regions A and B exhibit typical stress states within our sample material under impact loading conditions. Therefore, these two regions are of particular interest when evaluating the impact sensitivity of PBS.

#### 4.3.3. Analysis of Damage Evolution in PBS 

**Figure 19:**
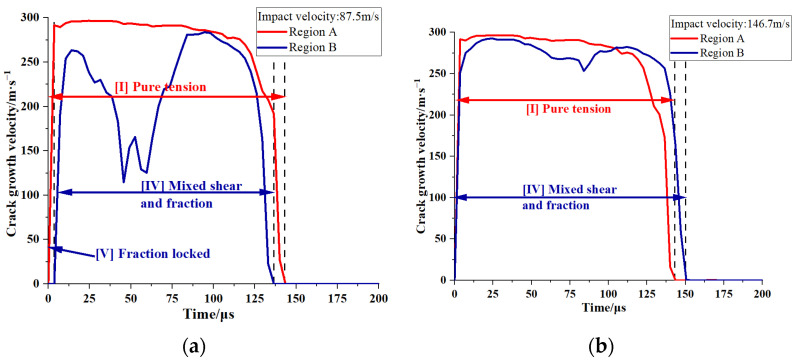
Crack growth velocity histories for regions A and B (corresponding to Figure 15) at impact velocities of (**a**) 87.5 m/s and (**b**) 146.7 m/s. The dashed line serves as a reference at the start and end of the curve.

Crack propagation on the $\sigma_{1}$–$\sigma_{3}$ plane is affected by different stress conditions, which can be classified into five groups: [I] pure tension; [II] a combination of tensile and shear stresses; [III] pure shear; [IV] a combination of shear and friction forces; and [V] friction locked. Figure 19 illustrates the historical crack growth velocities in regions A and B and elucidates the temporal changes in stress conditions. Following the initial impact, region A in the PBS exhibits a type I stress state (Figure 19a,b). Subsequently, opening cracks are formed, and their sizes increase substantially because of tensile effects. In contrast to region A, the cracks in region B remain stable during the initial 0–5 µs period at an impact velocity of 87.5 m/s (Figure 19a). This stability is attributed to the stress state being closely aligned with hydrostatic compression, which is characterized by the following conditions during this period: $\sigma_{3}$ ≤ $\sigma_{1}$ ≤ 0 and 1 ≤ $\frac{\sigma_{3}}{\sigma_{1}}$ ≤ ${\left(\sqrt{\mu_{s}^{2}+1}+\mu_{s}\right)}^{2}$. The interfacial friction consistently exceeds the applied shear force at the crack surface, resulting in the closure and stabilization of cracks in region B; this process is known as friction-locking. However, after 5 µs, the applied shear force becomes substantial enough to overcome this interfacial friction. Owing to the high stress concentration in region B, the applied load leads to crack instability and a rapid increase in average crack size. Figure 19a demonstrates that an impact velocity of 87.5 m/s causes a type V-to-type IV stress state transition at 5 µs. Conversely, Figure 19b illustrates that an impact velocity of 146.7 m/s consistently results in a type IV stress state owing to the incident stress wave.

**Figure 20:**
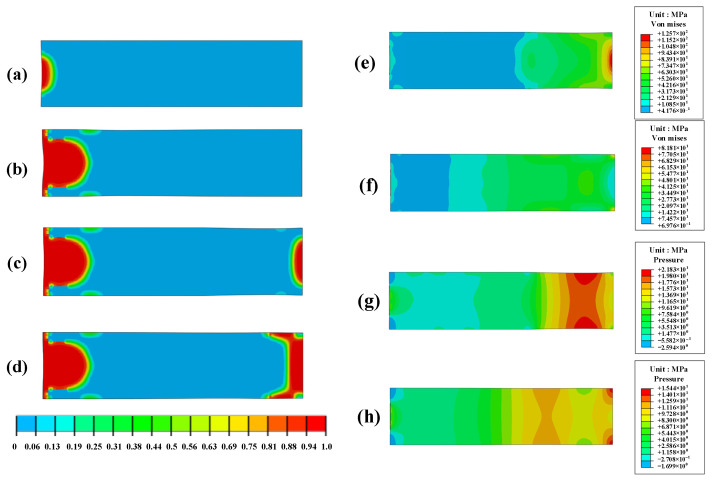
Microcrack damage contours of PBS with 87.5 m/s impact velocity (**a**) 10 µs, (**b**) 140 µs, (**c**) 262 µs, and (**d**) 350 µs; Von Mises stress contours of PBS with 87.5 m/s impact velocity; (**e**) 262 µs, and (**f**) 350 µs. Pressure contours of PBS with 87.5 m/s impact velocity (**g**) 262 µs, and (**h**) 350 µs.

To evaluate the macroscopic damage evolution of PBS, Figure 20a–d illustrates the contours of damage fraction at an impact velocity of 87.5 m/s at *t* = 70 µs, 140 µs, 262 µs, and 350 µs. The damage fraction (*D*) can be categorized into four levels [44]: level 1 (0.3 ≤ *D* ≤ 0.5), level 2 (0.5 < *D* ≤ 0.75), level 3 (0.75 < *D* ≤ 0.9), and level 4 (0.9 < *D* ≤ 1.0) represent slightly damaged, moderately damaged, severely damaged, and completely fractured PBS samples, respectively. During the initial loading stage, region A undergoes severe damage. As loading increases, a hemispherical region beneath the impact surface predominantly exhibits damage at *t* = 140 µs. 

Because stress waves propagate through the sample, both damaged and fractured areas tend to expand, eventually encompassing nearly all parts of the sample. This expansion enlarges the opening cracks. When incident stress waves reach the bottom of the PBS shell interface under different wave impedances, rarefaction waves are generated due to the reflection at this interface, resulting in stress concentration, as shown in Figure 20e,f. Owing to these rarefaction wave effects, the bottom part of PBS is damaged (Figure 20c,d). The stress level inside PBS decreases with decreasing external loads, as depicted in Figure 20f,h. This pattern aligns well with the experimental findings, thus validating the reliability of both the subroutine and computational process.

## 5. Conclusions

This paper proposed PBS as a viable alternative to PBX. A constitutive model incorporating multiple stress-state-motivated evolution modes of microcracks was developed to examine the overall damage characteristics of PBS.

When the PBS shell was loaded with a first-stage light gas gun, the stress peak increased with increasing impact velocity, while the attenuation characteristics exhibited an upward trend over greater distances. After penetration, a cone-shaped pit was formed in the PBS shell, and the penetration depth was proportional to impact velocity. SEM analysis revealed that damage levels gradually decreased along the axial direction; however, severe damage and macro cracks were evident near the impact surface.

The viscoelastic damage constitutive model and the developed VUMAT subroutine were employed to analyze the damage behavior of the PBS. The damage was primarily concentrated in an annular pattern at both the center and edge of the direct impact surface. Moreover, the high compression region (specifically near the sample’s center) exhibited considerable damage under shear sliding, while rapid microcrack propagation led to severe damage near the impact surface. As incident stress waves propagated through PBS toward the bottom, rarefaction waves were generated after reflection at the interface because of differences in wave impedance between PBS and shell materials. Consequently, these rarefaction waves induced additional damage at the bottom of PBS. Future studies must evaluate the mechanical properties of PBS, including micro-void collapse/distortion, crack statistics, including mean size and number density, and specific surface energy to improve the predictive accuracy of our model.

## Figures and Tables

**Table 1:** PBS composition.

Sugar (wt.%)	HTPB (wt.%)	DOS (wt.%)	TDI (wt.%)
90	7.2	1.593	1.207

**Table 2:** Shear modulus and relaxation parameter for PBS.

*Parameter*	Value	*Parameter*	Value
1/*τ*_1_	7.32	*G*_1_ (MPa)	353.8
1/*τ*_2_	73.2	*G*_2_ (MPa)	497.2
1/*τ*_3_	732.1	*G*_3_ (MPa)	597.5
1/*τ*_4_	7320.6	*G*_4_ (MPa)	660.5
1/*τ*_5_	0	*G*_5_ (MPa)	744.0

**Table 3:** Cracking parameters for PBS.

*Parameter*	Value	*Parameter*	Value
*c* (mm)	0.03	*V_R_* (m/s)	300.0
*a* (mm)	1.0	*m*	6.0
*ν*	0.48	*Γ*	5.0 × 10^−5^
*ρ* (g/cm^3^)	1.49	*μ*_*s*_	660.5

**Table 4:** Johnson-Cook parameters for 4340 steel.

*Parameter*	Value	*Parameter*	Value	*Parameter*	Value	*Parameter*	Value
*ρ* (g/cm^3^)	7.85	*ν*	0.3	*B* (MPa)	510.0	*c*	0.014
*E* (GPa)	210.0	*A* (MPa)	792.0	*n*	0.26	*m*	1.03

## Data Availability

The data presented in this study are available on request from the corresponding author.
